# Shared diagnostic genes and potential mechanisms between polycystic ovary syndrome and recurrent miscarriage revealed by integrated transcriptomics analysis and machine learning

**DOI:** 10.3389/fendo.2024.1335106

**Published:** 2024-09-27

**Authors:** Juanjuan He, Ahui Liu, Haofei Shen, Yanbiao Jiang, Min Gao, Liulin Yu, Wenjing Du, Xuehong Zhang, Fen Fu

**Affiliations:** ^1^ The Second Affiliated Hospital, Jiangxi Medical College, Nanchang University, Nanchang, Jiangxi, China; ^2^ The First School of Clinical Medicine, Lanzhou University, Lanzhou, Gansu, China; ^3^ The First Hospital of Lanzhou University, Lanzhou, Gansu, China

**Keywords:** PCOS, RSA, bioinformatics analysis, co-diagnostic genes, mechanism research, immune infiltration

## Abstract

**Objective:**

More and more studies have found that polycystic ovary syndrome (PCOS) is significantly associated with recurrent spontaneous abortion (RSA), but the specific mechanism is not yet clear.

**Methods:**

Based on the GEO database, we downloaded the PCOS (GSE10946, GSE6798 and GSE137684) and RSA (GSE165004, GSE26787 and GSE22490) datasets and performed differential analysis, weighted gene co-expression network (WGCNA), functional enrichment, and machine learning, respectively, on the datasets of the two diseases, Nomogram and integrated bioinformatics analysis such as immune infiltration analysis. Finally, the reliability of the diagnostic gene was verified by external verification and collection of human specimens.

**Results:**

In this study, PCOS and RSA datasets were obtained from Gene Expression Omnibus (GEO) database, and a total of 23 shared genes were obtained by differential analysis and WGCNA analysis. GO results showed that the shared genes were mainly enriched in the functions of lipid catabolism and cell cycle transition (G1/S). DO enrichment revealed that shared genes are mainly involved in ovarian diseases, lipid metabolism disorders and psychological disorders. KEGG analysis showed significant enrichment of Regulation of lipolysis in adipocytes, Prolactin signaling pathway, FoxO signaling pathway, Hippo signaling pathway and other pathways. A diagnostic gene FAM166 B was obtained by machine learning and Nomogram screening, which mainly played an important role in Cellular component. GSEA analysis revealed that FAM166B may be involved in the development of PCOS and RSA by regulating the cell cycle, amino acid metabolism, lipid metabolism, and carbohydrate metabolism. CIBERSORT analysis showed that the high expression of FAM166 B was closely related to the imbalance of multiple immune cells. Further verification by qPCR suggested that FAM166 B could be used as a common marker of PCOS and RSA.

**Conclusions:**

In summary, this study identified FAM166B as a common biomarker for PCOS and RSA, and conducted in-depth research and analysis of this gene, providing new data for basic experimental research and early prognosis, diagnosis and treatment of clinical diseases.

## Introduction

Polycystic ovary syndrome (PCOS) has become one of the most common and serious endocrine diseases affecting the reproductive health of women of childbearing age in the 21st century, the prevalence rate is as high as 15%, which is mainly characterized by hyperandrogenism (HA), ovarian dysfunction (oligoovulation or anovulation) and polycystic ovary morphology (PCOM), irregular menstruation, infertility and excessive androgen (hirsutism and acne) are the main clinical symptoms of PCOS (1–3). It is worth noting that infertility is currently the primary problem plaguing women of childbearing age. Although many infertile women with PCOS can be pregnant through assisted reproductive technology (ART) treatment, the risk of pregnancy also increases (4), such as recurrent spontaneous abortion, repeated implantation failure, insulin resistance (IR), gestational diabetes, gestational hypertension, etc. (5, 6), which seriously affect the pregnancy ability of women of childbearing age, among them, recurrent spontaneous abortion is one of the main concerns of scholars in recent years (7).

Recurrent spontaneous abortion (RSA) refers to two or more spontaneous abortions with the same partner before 20-24 weeks of pregnancy (8, 9), affecting 1-5% of pregnant women (10), it is often related to anatomical defects, chromosomal abnormalities, immune microenvironment, endocrine disorders and psychological factors (11, 12). In recent years, the relationship between PCOS, IR and RSA has gradually attracted the attention of many scholars (13). It is reported that about 70% of PCOS women have IR (14), and about 40% of RSA women have PCOS (15), this suggests that polycystic ovary syndrome and RSA may have a common pathophysiological process (16–18). Unfortunately, the current research on the potential mechanism between PCOS and RSA disease is still inconclusive, and few studies have focused on the common genetic characteristics and molecular mechanisms of PCOS and RSA.

In this study, we integrated PCOS and RSA-related transcriptomic data from the Gene Expression Omnibus (GEO) database, and applied the “limma” package and weighted gene co-expression network analysis (WGCNA) to identify differentially expressed genes and key modules in each disease, and performed enrichment analysis of shared genes. Subsequently, the target gene was screened by three kinds of machine learning and Nomogram, and a common diagnostic gene FAM166B was finally identified. Next, we performed functional enrichment analysis of FAM166B to determine the common pathways associated with PCOS and RSA. In addition, we learned about the regulatory role of FAM166B on immune cells in two diseases through CIBERSORT. Finally, the reliability of FAM166 B was verified by external data sets and human samples.

In conclusion, this study provides valuable insights into the common molecular mechanisms of PCOS and RSA, and highlights the potential of FAM166B as a diagnostic marker for PCOS and RSA, laying the foundation for targeted therapy and improving the fertility of PCOS patients.

## Materials and methods

### Collection and processing of data sets

In this study, data sets related to PCOS and RSA were retrieved in GEO (http://www.ncbi.nih.gov/geo/) database (19), and the inclusion criteria were set as: (1) Homo sapiens; (2) Detection of RNA expression profile by gene chip; (3) disease group and control group included; (4) Each dataset contains at least 10 samples. Finally, three PCOS datasets (GSE10946, GSE6798, GSE137684) and three RSA datasets (GSE165004, GSE26787, GSE22490) were retrieved and included in the study. GSE10946 and GSE165004 were used for the analysis and verification of PCOS and RSA. GSE6798 and GSE137684 of PCOS, GSE26787 and GSE22490 of RSA were used for external validation. Detailed information about the dataset is shown in **Table 1**.

**Table 1:** Information of GSE Datasets.

Diseases	Datasets	Platform	Species	Total sample	Data type
PCOS	GSE10946	GPL570	Homo sapiens	23	Microarray
	GSE6798	GPL570	Homo sapiens	29	Microarray
	GSE137684	GPL17077	Homo sapiens	12	Microarray
RSA	GSE165004	GPL16699	Homo sapiens	48	Microarray
	GSE26787	GPL570	Homo sapiens	10	Microarray
	GSE22490	GPL570	Homo sapiens	10	Microarray

The downloaded gene expression matrix files were read, annotated, corrected, merged and batch effects eliminated using the “limma” and “sva” packages to obtain standardized gene expression data for analysis.

### Differential gene expression analysis

We used the “limma” package to obtain differentially expressed genes (DEGs) in the PCOS and RSA groups. The DEGs standard of PCOS group was set as *P* < 0.05 and |log2FC|>0.25; RSA used *P* < 0.05 and |log2 FC|>0.65 as the standard for screening DEGs. The DEGs of the two groups were visualized by the volcano map and heat map made by the “ggplot2” and “pheatmap” package.

### Weighted gene co-expression network analysis

Weighted gene co-expression network analysis (WGCNA) is a sophisticated systems biology method for elucidating patterns of association between genes in microarray samples. The method helps to identify sets of genes with strong co-variation and reveal potential candidate biomarker genes or therapeutic targets by exploring the intrinsic linkages within the set of genes and their correlation with the phenotype (20). In this study, the top 5000 genes with large variance were selected from the normalized mRNA expression data for WGCNA analysis, and the “gplots” package was used for hierarchical clustering analysis. The selection of a soft threshold power (β) was determined by applying the pickSoftThreshold function and adhering to the scale-free topology criterion, and the β is mainly related to the independence and average connectivity of the co-expression module. Topological overlap degree (TOM) represents the overlap degree of network neighbors, and (1-TOM) retrieves pairwise distances to identify hierarchical clustering nodes and modules (21). The dynamic tree cutting algorithm of “pheatmap” package (minModuleSize=100, mergeCutHeight= 0.25) was used to obtain the gene modules related to the disease group.

### Identification of shared genes and functional enrichment analysis

After variance analysis and WGCNA analysis, 23 common genes were obtained in the PCOS and RSA groups, and in order to better understand the biological functions of these common genes, in this study, we used the “clusterProfiler” software package to perform enrichment analysis of the Gene Ontology (GO) [which includes Cellular Component (CC), Biological Process (BP) and molecular function (MF)], disease ontology (DO) and Kyoto Encyclopedia of the Genome (KEGG) were enriched and analyzed (22, 23), with *P* < 0.05 indicating statistical significance. The “enrichplot”, “circlize” and “ggplot2” software packages were used for data visualization.

### Machine learning algorithms to screen potential common markers

In this study, three mature machine learning algorithms are used, including least absolute shrinkage and selection operator (LASSO), support vector machine recursive feature elimination (SVM-RFE), and random forest (RF). In order to ensure the repeatability of these algorithms, we set the seeds to 12345 (24).

Firstly, shared genes were input into the LASSO algorithm, and the regression model was constructed using the “glmnet” package, and 10-fold cross-validation was carried out. In the “family” parameter, we set the “binomial”. The choice of λ has an important impact on the results of Lasso regression. If λ is too small, the regularization is not strong enough, which may lead to overfitting of the model; if λ is too large, the regularization is too strong, which may lead to underfitting of the model, which is generally chosen to be the minimum standard value. In this study, lambda.min was used as the best lambda value, and the shrinkage coefficient diagram of LASSO regression variable and the relationship diagram between LASSO regression model error and log (λ) were drawn.

Then, the random forest model is constructed using the “randomforest” package. The random forest model is used to determine the optimal number of variables by calculating the average error rate of candidate genes. In this study, error rates were calculated for each of the trees from 1 to 500, and the optimal number of trees and each candidate diagnostic gene feature importance score were determined based on the lowest error rate, with the top 10 genes in terms of importance being used for subsequent analyses.

Finally, the SVM-RFE model was constructed using the “caret” package and the “e1071” package, and was cross-validated with 10 folds to obtain the set of genes with the lowest 5×CV error and the highest 5×CV accuracy, and this set of genes was considered to be the relatively accurate diagnostic genes.

### Nomogram

In this study, the result variables of the final screening of machine learning are used as predictors, and a nomogram model is constructed through the “rms” package (25). “Points” represents the contribution score of each factor, and “Total Points” refers to the total score of all factors. In order to verify the performance of the nomogram, the consistency index (C-index) is calculated, and the discrimination is evaluated by the bootstrap method of 1000 resampling. Then, calibration curves were plotted to observe the relationship between the ideal diagnostic rate and the actual diagnostic rate obtained from the nomogram. The receiver operating characteristic (ROC) curve is used to assess the diagnostic performance of the genetic characterization model. The ROC analysis generated the area under the curve (AUC) and 95% confidence interval (CI), and an AUC value > 0.7 was considered to have great diagnostic efficacy.

### Identification and validation of shared diagnostic genes

In this study, 2 datasets from PCOS (GSE6798 and GSE137684) and RSA (GSE26787 and GSE22490) were used for external validation, with PCOS containing 41 samples (17 control samples and 24 experimental samples) and RSA containing 20 samples (11 control samples and 9 experimental samples). The PCOS and RSA datasets were read, annotated, corrected, merged, and batch effects eliminated using the “limma” and “sva” packages, resulting in standardized gene expression data for analysis. Box plots and ROC curves of the “limma”, “ggpubr” and “pROC” packages were used to understand the expression patterns and diagnostic performance of the co-diagnostic genes.

### Shared gene markers and functional enrichment analysis

Basic information about FAM166B was obtained through the Uniprot online platform (https://www.uniprot.org/). To understand the biological functions in which the shared markers are mainly involved, we performed GO enrichment and GSEA enrichment of the FAM166B gene using the “clusterProfiler” package, and visualized the biological signaling pathways associated with PCOS and RSA using the “enrichplot” package and “pathview” package.

### Gene set variation analysis

GSVA is a non-parametric and unsupervised algorithm, which transforms the change of gene level into the change of pathway level by comprehensively scoring the gene set of interest, and then judges the biological function of the sample (26). In this study, gene sets (c2.cp.kegg.Hs.symbols.gmt) were downloaded from the Molecular Characterization Database, and the GSVA algorithm was used to score each gene set comprehensively, thus assessing potential biological functional changes in different samples.

### Immune cell infiltration

The CIBERSORT algorithm analyses the composition of immune cells based on normalized gene expression profiling data via deconvolution (27, 28). The LM22 in CIBERSORT website (http://cibersort.stanford.edu/) contains 22 annotated genetic features. Based on the genetic characterization of LM22, this study quantified 22 immune cells by the CIBERSORT algorithm and 1000 iterations to understand the distribution of immune cells in the two disease groups. Subsequently, the correlation between immune cells and diagnostic genes was analyzed. Finally, the results are visualized using the “corplot” package, the “vioplot” package, and the “ggpubr” package.

### Collection of clinical tissue samples

To further validate the expression of FAM166B in PCOS and RSA, granulosa cells were collected from six patients who underwent *in vitro* fertilization-embryo transfer (IVF-ET)/Intracytoplasmic sperm injection (ICSI) at the Reproductive Center of the First Affiliated Hospital of Lanzhou University (Lanzhou, China) in the present study, of which three were due to infertility due to PCOS and 3 due to male factor or tubal factor. Inclusion criteria for PCOS (1): fulfillment of the diagnostic criteria for PCOS initiated by ESHRE/ASRM Rotterdam (29); (2) age 20-40 years. Control group: (1) patients with infertility due to male factor or tubal factor; (2) age 20-40 years old. Exclusion criteria: (1) age greater than 40 years; (2) mental illness or inability to communicate normally; (3) chromosomal abnormalities in one or both spouses; (4) exclusion of endocrine diseases and cardiovascular and cerebral vascular diseases; (5) organic disorders of the uterus and ovaries, gonadal insufficiency, etc.; and (6) history of antibiotic treatment and autoimmune disorders in the past 3 months. In this study, the effect of ovulation promotion regimen on the outcome of the study was considered, and all the included populations were subjected to a progestin primed ovarian stimulation (PPOS) regimen with high progesterone. When participants’ vaginal ultrasound monitoring showed 3 or more follicles growing to≥18 mm in diameter, appropriate amounts of human chorionic gonadotrophin (HCG) were administered as appropriate, and oocytes were collected 36 hours after administration under vaginal ultrasound guidance. Granulosa cells (GCs) from multiple pooled follicles per subject were collected from approximately 20 mL of follicular fluid using sterile test tubes and separated using Ficoll-Percoll (Solarbio-Life-Sciences, Beijing, China), labeled and loaded into 1.5 mL centrifuge tubes, which were immediately placed in - 80°C refrigerator for freezing and storage for subsequent RT-qPCR.

In addition, chorionic villus tissue samples from four women with RSA and four women with elective termination of pregnancy were collected to validate the expression of FAM166B in RSA. Inclusion criteria for the RSA group were (1) history of two or more unexplained miscarriages and (2) absence of fetal heartbeat by ultrasound at 6-8 weeks of gestation. Control group: (1) natural pregnancy of 6-8 weeks duration and voluntary termination of pregnancy for non-medical reasons; (2) No symptoms of threatened abortion; (3) fetal cardiac activity observed by ultrasound within 3 days before termination of pregnancy. Exclusion criteria: (1) karyotyping of abnormal embryos; (2) known causes of miscarriage; and (3) patients with other concurrent medical conditions. After the patient had an abortion, the chorionic tissue was transferred to a curved disk with forceps within 15 minutes, the tissue was rinsed thoroughly with saline, the surrounding blood clot was removed, and a sample of approximately 4 g was separated with a scalpel and placed in a cryotube with RNA cryopreservative solution and stored in a refrigerated tube at -80°C for use in subsequent RT-qPCR.

The study was approved by the Ethics Committee of the First Affiliated Hospital of Lanzhou University, Lanzhou, China (Ethics No. LDYYSZLL2023-25) and complied with the principles of the Declaration of Helsinki.

### RT-qPCR

Total RNA was extracted from tissues with Trizol (Takara, Japan) according to the instructions of the Total RNA Extraction Kit (Coolaber, RE600). RNA purity and concentration were determined using a NanoDrop 2000 (Thermo Fisher Scientific, USA). SweScript All-in-One First Strand cDNA Synthesis Kit (Servicebio, G3337) reverse transcribed total RNA into cDNA. The PCR reaction was performed using SYBR Green Master Mix kit from Qiagen, Germany, with cDNA as template and human GAPDH as internal reference. The primers were designed and synthesized by Sevier Biotechnology Ltd. and the primer sequences are shown in **Supplementary Table 1**. qRT-PCR was performed as previously reported (30), and primer specificity was ensured by observing the melting curve of the reaction during qPCR. At least three biological replicates and three technical replicates were performed in this experiment, and the relative expression of mRNA of target genes was calculated using the 2-ΔΔCT method. Statistical analyses were performed using the Mann-Whitney U test, and data were expressed as mean and standard error of the mean (SEM). *P* < 0.05 was considered a statistically significant difference.

### Statistical analysis

Statistical analysis was performed using R (4.3.1) or GraphPad Prism (9.5.0), and *P* < 0.05 was considered statistically significant.

## Results

### Identification of DEGs

**Figure 1:**
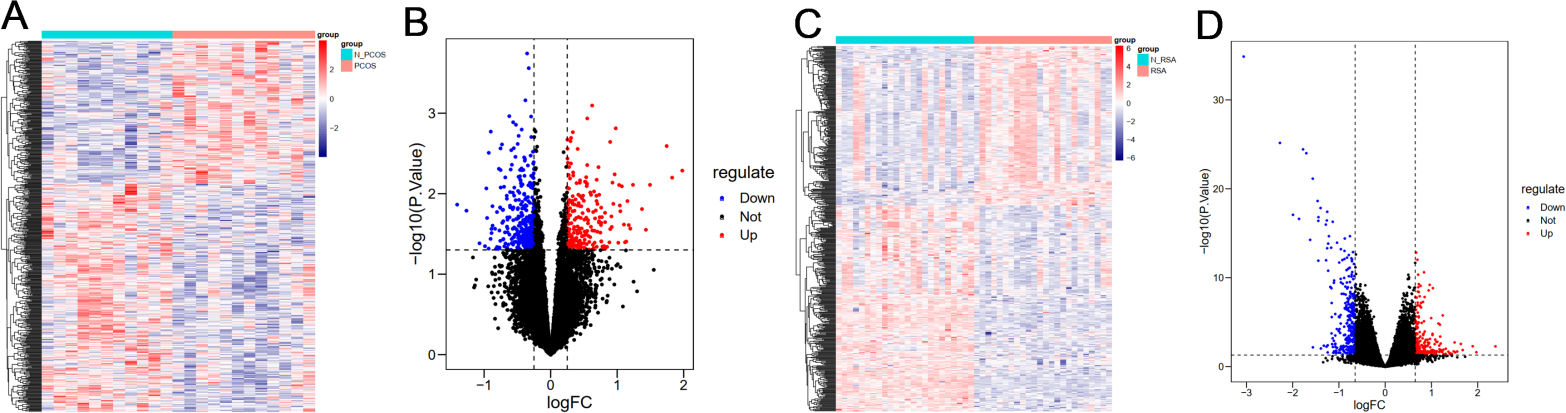
Visualization of differential genes. **(A)** Heatmap of DEGs in PCOS group. **(B)** Volcano plot of DEGs in PCOS group. **(C)** Heatmap of DEGs in RSA group. **(D)** Volcano plot of DEGs in RSA group.

DEGs for PCOS and RSA were analyzed using the “limma” package. A total of 537 DEGs were obtained from the PCOS group, of which 207 were up-regulated genes and 330 were down-regulated genes. A total of 517 DEGs were obtained from the RSA group, including 226 up-regulated genes and 291 down-regulated genes. Heat maps (**Figures 1A, C**) and volcano plots (**Figures 1B, D**) show that the distribution of the differential genes differed markedly between the disease and control groups, with red representing up-regulation and blue representing down-regulation.

### Screening for key gene modules by WGCNA

In order to study the key genes related to clinical phenotypes, in addition to analyzing the DEGs of the two groups, this study also constructed a co-expressed gene module. Cluster analysis showed that there was one outlier sample in the PCOS group, and it was removed. All samples in the RSA group are in the cluster, so all samples are retained.

**Figure 2:**
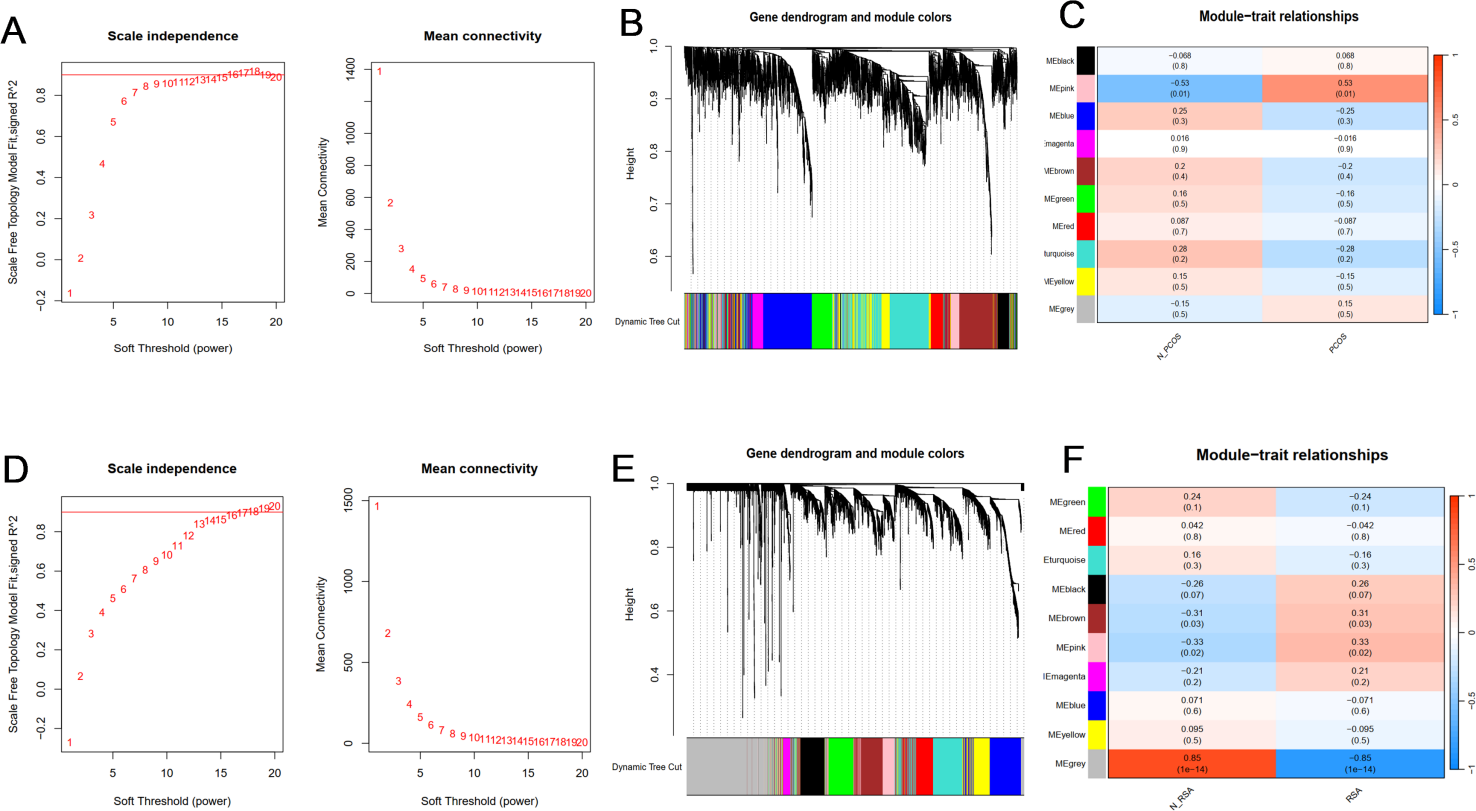
Construction and module analysis of WGCNA. **(A)** Determination of soft-threshold power for PCOS. **(B)** Cluster dendrogram of PCOS highly connected genes in key modules. **(C)** The correlation between co-expressed gene modules and clinical traits in PCOS. **(D)** Determination of soft-threshold power for RSA. **(E)** Cluster dendrogram of RSA highly connected genes in key modules. **(F)** The correlation between co-expressed gene modules and clinical traits in RSA. Different colors represent different co-expression modules. A correlation and *P* value are included in each cell.

According to the approximate scale-free topological standard, the β of the PCOS group model was set to 9 (**Figure 2A**), and the fitting index was set to 0.9; the adjacency matrix is generated by using the adjacency function, and the hierarchical clustering is constructed by using the TOM dissimilarity measure. After merging similar gene modules, 10 modules were found in the PCOS model, among which the MEpink module had the strongest positive correlation with PCOS (R = 0.53, *P* = 0.01), containing a total of 259 genes (**Figures 2B, C**). The maximum β of RSA group was 14 (**Figures 2D**), and the fitting index was set to 0.9.A total of 10 co-expression modules were identified, among which the grey module was strongly negatively correlated with the occurrence of RSA (R = 0.85, *P* < 0.001) (**Figures 2E, F**). These genes in the two key modules may be used as candidate markers.

### Shared gene and functional enrichment analysis

**Figure 3:**
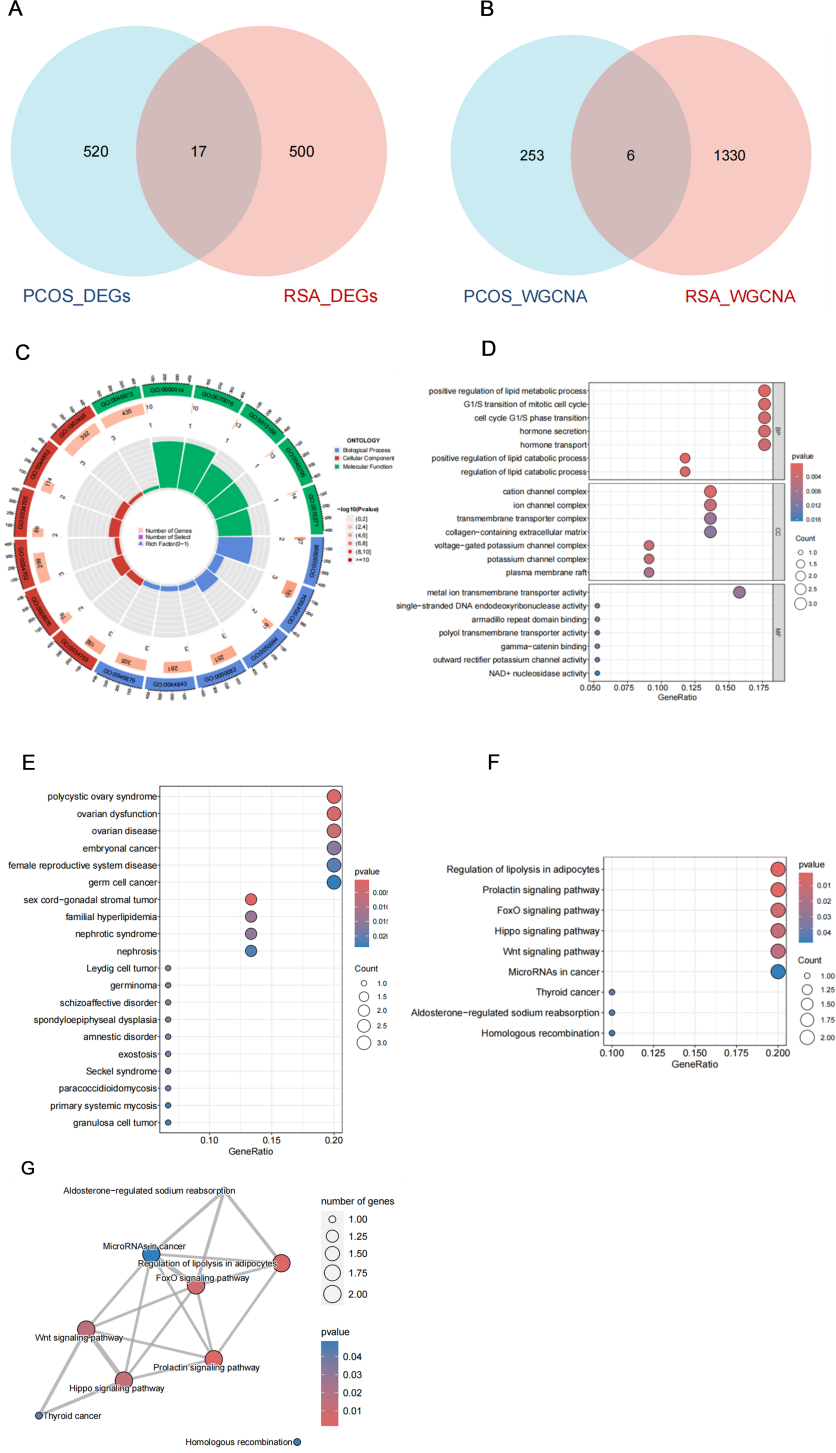
Shared gene characteristics and functional enrichment between PCOS and RSA. **(A)** The DEGs intersection genes of PCOS group and RSA group. **(B)** WGCNA module overlapping genes of PCOS and RSA. **(C)** GO enrichment chord graph of shared genes. **(D)** GO enrichment bubble diagram of shared genes. **(E)** DO enrichment bubble diagram of shared genes. **(F)** KEGG enrichment bubble diagram of shared genes. **(G)** KEGG enrichment network map of shared genes.

In order to explore the common mechanism of PCOS and RSA, we regard the intersection genes of DEGs and WGCNA as common candidate diagnostic genes (**Figures 3A, B**), and try to understand the potential biological changes between PCOS and RSA through functional annotation and enrichment analysis.

The GO enrichment results included Cellular Components (CC), Biological Process (BP) and Molecular Function (MF) (**Figure 3C**), of which the main enrichment in molecular biological function was in the regulation of lipid catabolic process and cell cycle G1/S phase transition; In Cellular Components, it was mainly involved in cation channel complex and ion channel complex; In addition, it is significantly enriched in metal ion transmembrane transporter activity, single-stranded DNA endodeoxyribonuclease activity, armadillo repeat domain binding, and other biological processes domain binding (**Figure 3D**). To further understand which diseases these genes are mainly involved in, we performed DO enrichment analysis, which showed that the common genes were mainly enriched in female reproductive system, lipid metabolism disorder-related diseases and psychological disorders, including polycystic ovary syndrome, germ cell cancer, germinoma, familial hyperlipidemia and schizoaffective disorder, etc. (**Figure 3E**). In the KEGG enrichment results, we noticed a significant enrichment of Regulation of lipolysis in adipocytes, Prolactin signaling pathway, FoxO signaling pathway, Hippo signaling pathway, Wnt signaling pathway, and other pathways were significantly enriched (**Figure 3F**), and these pathways collaborated closely with each other (**Figure 3G**).

### Screening potential co-diagnostic genes based on machine learning

In order to further screen out the most candidate diagnostic genes that have important contribution value to the classification of disease group and control group, based on the 23 common genes obtained from DEGs and WGCNA results, three different algorithms (LASSO, SVM-RFE and RF) were used for screening.

**Figure 4:**
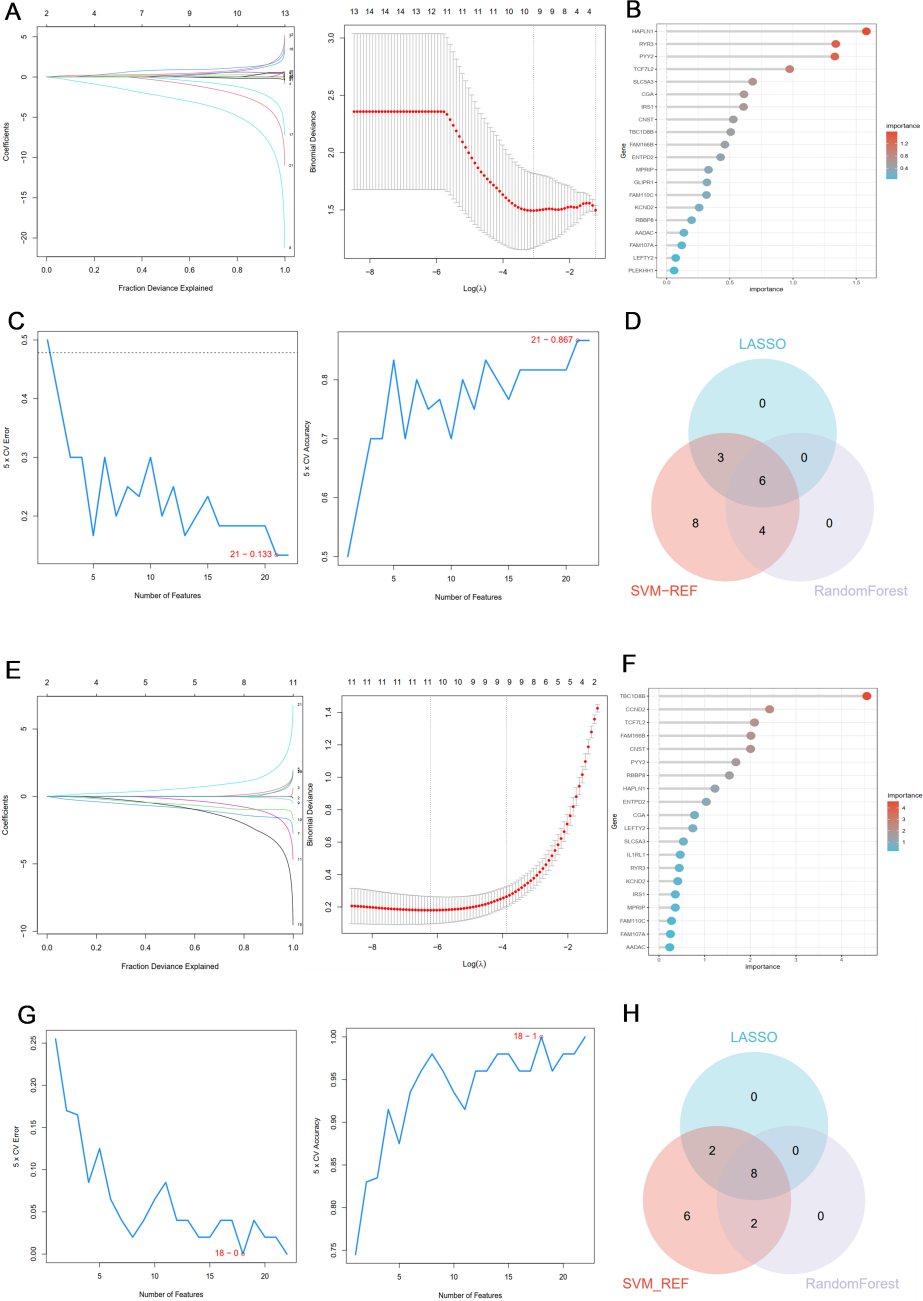
**(A)** Coefficient profile plot of the LASSO model for PCOS showed the final parameter selection λ (lambda). **(B)** The top 20 genes in the RF analysis results of the PCOS group. **(C)** SVM-RFE algorithm was used to screen 21 important genes for PCOS group. **(D)** Six candidate diagnostic genes in PCOS group. **(E)** Coefficient profile plot of the LASSO model for RSA showed the final parameter selection λ (lambda). **(F)** The top 20 genes in the RF analysis results of the RSA group. **(G)** SVM-RFE algorithm was used to screen out 18 important genes for RSA group. **(H)** Eight candidate diagnostic genes in RSA group.

In the PCOS group, the λ of Lasso was set to 0.05051258 (**Figure 4A**), and 9 genes with non-zero coefficients were identified; the top 10 genes in the random forest results were selected (**Figure 4B**); the SVM algorithm was used to identify the 21 genes with the smallest error (**Figure 4C**); finally, the Venn algorithm was used to intersect the three results, and six candidate markers (RYR3, TBC1D8B, CNST, FAM166B, SLC5A3, PYY2) were identified for the PCOS group (**Figure 4D**). Similarly, in the RSA group, when the λ of the LASSO algorithm was set to 0.004239023, 10 characteristic genes were obtained (**Figure 4E**); the top 10 genes of random forest results were selected (**Figure 4F**); the SVM-REF algorithm was used to determine the 18 hub genes with the smallest error (**Figure 4G**); finally, eight common genes (TBC1D8B, HAPLN1, CCND2, TCF7L2, FAM166B, RBBP8, CGA, PYY2) obtained by Venn analysis were identified as potential markers for the diagnosis of RSA (**Figure 4H**).

### Nomogram of candidate markers

**Figure 5:**
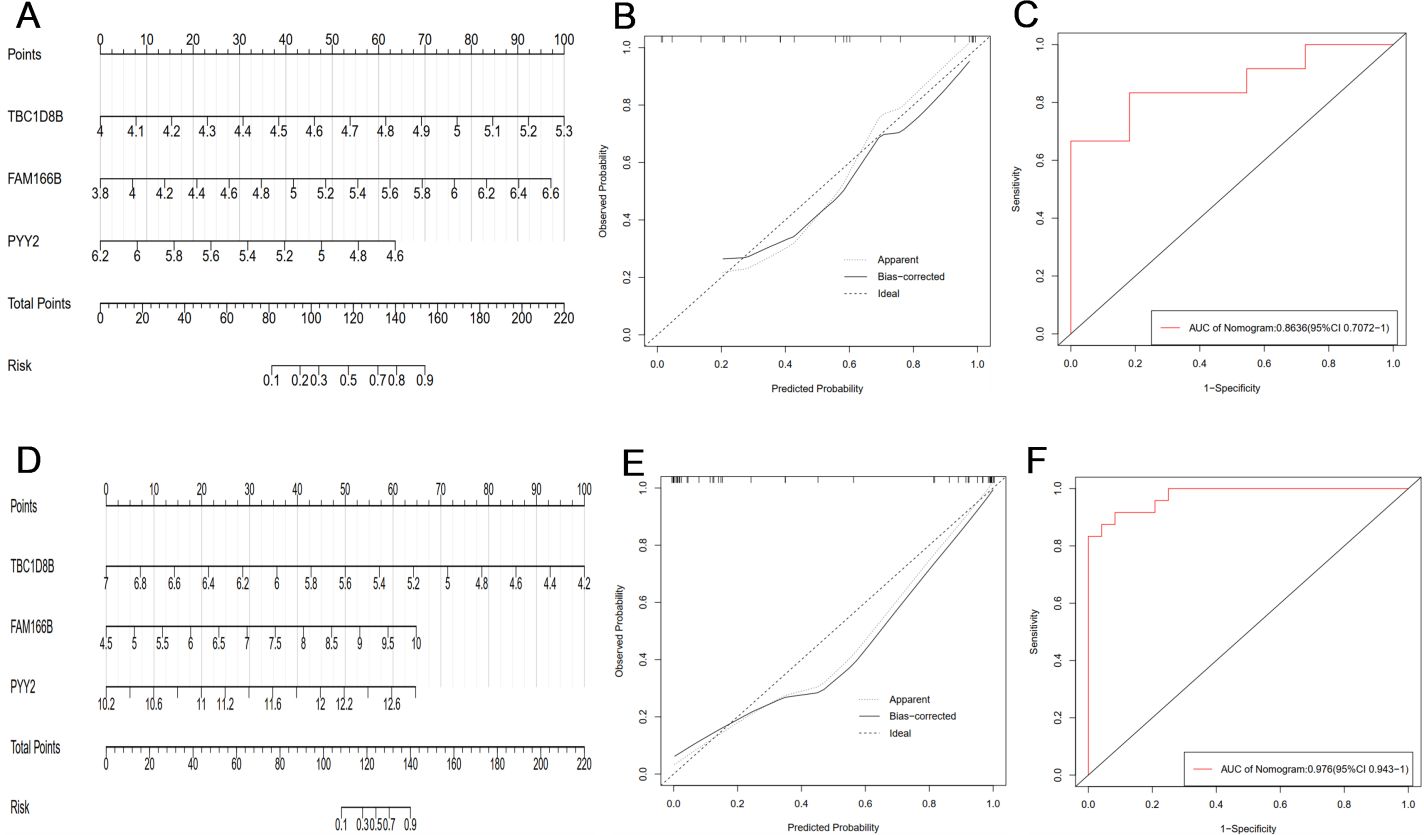
**(A)** The PCOS group constructed a nomogram model based on three key genes (TBC1D8B, FAM166B, PYY2). **(B)** The calibration curve of PCOS nomogram model. **(C)** ROC curve of PCOS group nomogram model. **(D)** The RSA group constructed a nomogram model based on three key genes (TBC1D8B, FAM166B, PYY2). **(E)** Calibration curve of RSA nomogram model. **(F)** The ROC curve of the RSA group nomogram model.

In order to further determine the common diagnostic genes of PCOS and RSA, we used the cross genes of two sets of machine learning results (TBC1D8B, FAM166B, PYY2) as predictors to construct two sets of nomogram models (**Figures 5A, D**). The calibration curves showed that the actual diagnostic rates of the two models were more similar to the ideal diagnostic rates, indicating that the column-line plots had a better predictive value, and the mean absolute errors of the PCOS and RSA models were 0.053 and 0.043, respectively (**Figures 5B, E**). In addition, the AUC of the PCOS group nomogram model was 0.864 (95% CI 0.707-1) (**Figure 5C**), and the AUC of the RSA group nomogram model was 0.976 (95% CI 0.943-1) (**Figure 5F**). The overall results showed that the two sets of prediction models had fair accuracy and discrimination, and the three key genes had high clinical value in the diagnosis of PCOS and RSA.

### Identification and verification of shared diagnostic genes

**Figure 6:**
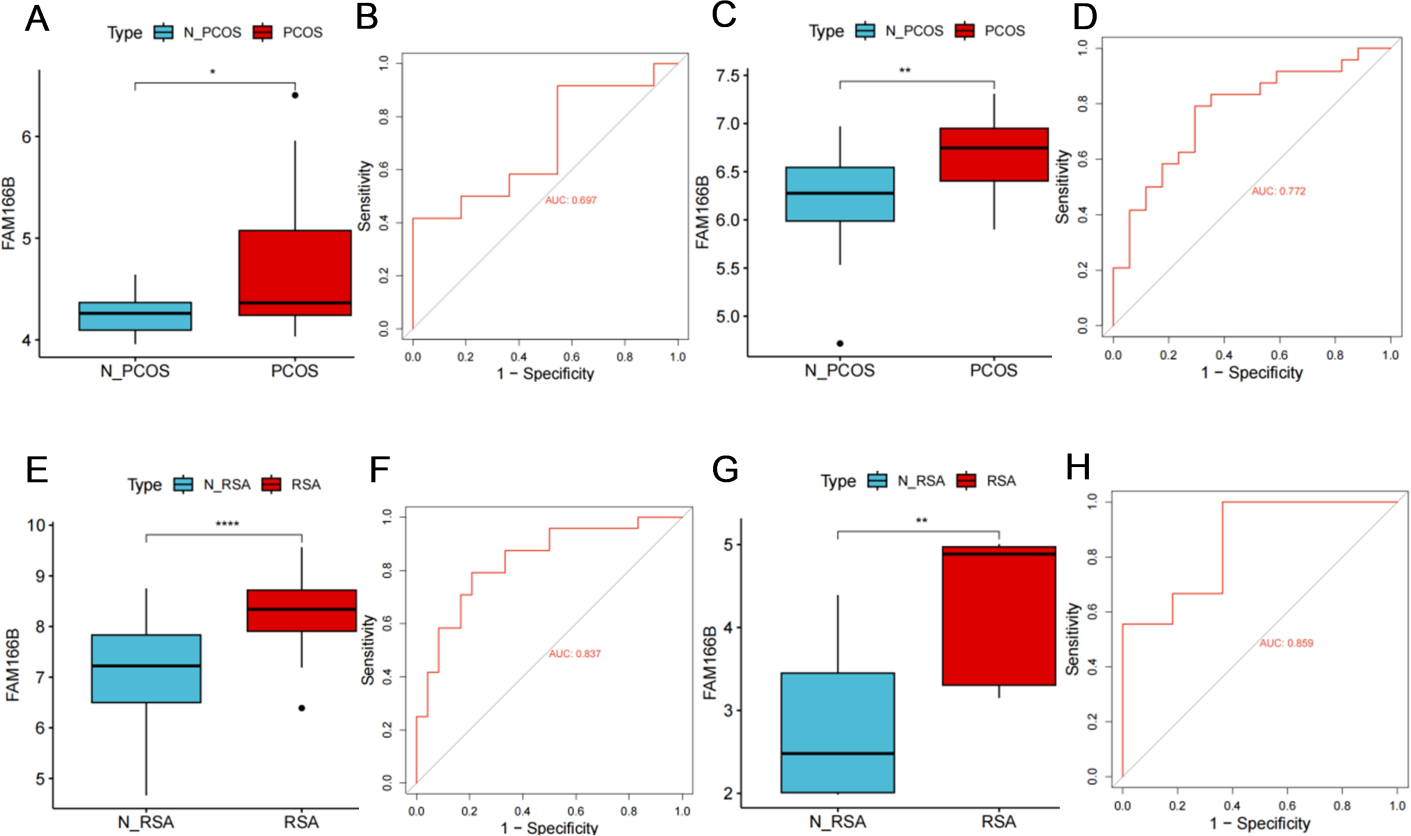
**(A)** The differential expression of FAM166 B in PCOS training groups. **(B)** ROC curve of FAM166 B in PCOS training group. **(C)** The differential expression of FAM166 B in PCOS validation group. **(D)** ROC curve of FAM166 B in PCOS validation group. **(E)** The differential expression of FAM166 B in RSA training group. **(F)** ROC curve of FAM166 B in RSA training group. **(G)** The differential expression of FAM166 B in RSA validation group. **(H)** ROC curve of FAM166 B in RSA validation group. *P <0.05, **P < 0.01, ****P<0.0001.

In order to identify the key genes with diagnostic value for PCOS and RSA, the expression patterns and receiver operating characteristic curve (ROC curve) of three candidate diagnostic genes (TBC1D8B, FAM166B, PYY2) were analyzed in this study. Finally, FAM166B was identified as the best shared diagnostic gene. It is worth noting that the expression of FAM166 B was significantly increased in PCOS and RSA (**Figures 6A, E**) (*P* < 0.05), and the prediction performance was relatively robust (AUC = 0.697, 0.772) (**Figures 6B, F**).

In addition, in the external validation cohorts of PCOS and RSA, the expression level of FAM166B was consistent with that of the training group (**Figures 6C, G**), and had higher diagnostic performance in the validation cohorts of PCOS and RSA (AUC = 0.772, 0.859) (**Figures 6D, H**). In summary, various results have confirmed the reliability of FAM166B as a biomarker for the diagnosis of PCOS and RSA.

### Functional enrichment of FAM166B

**Figure 7:**
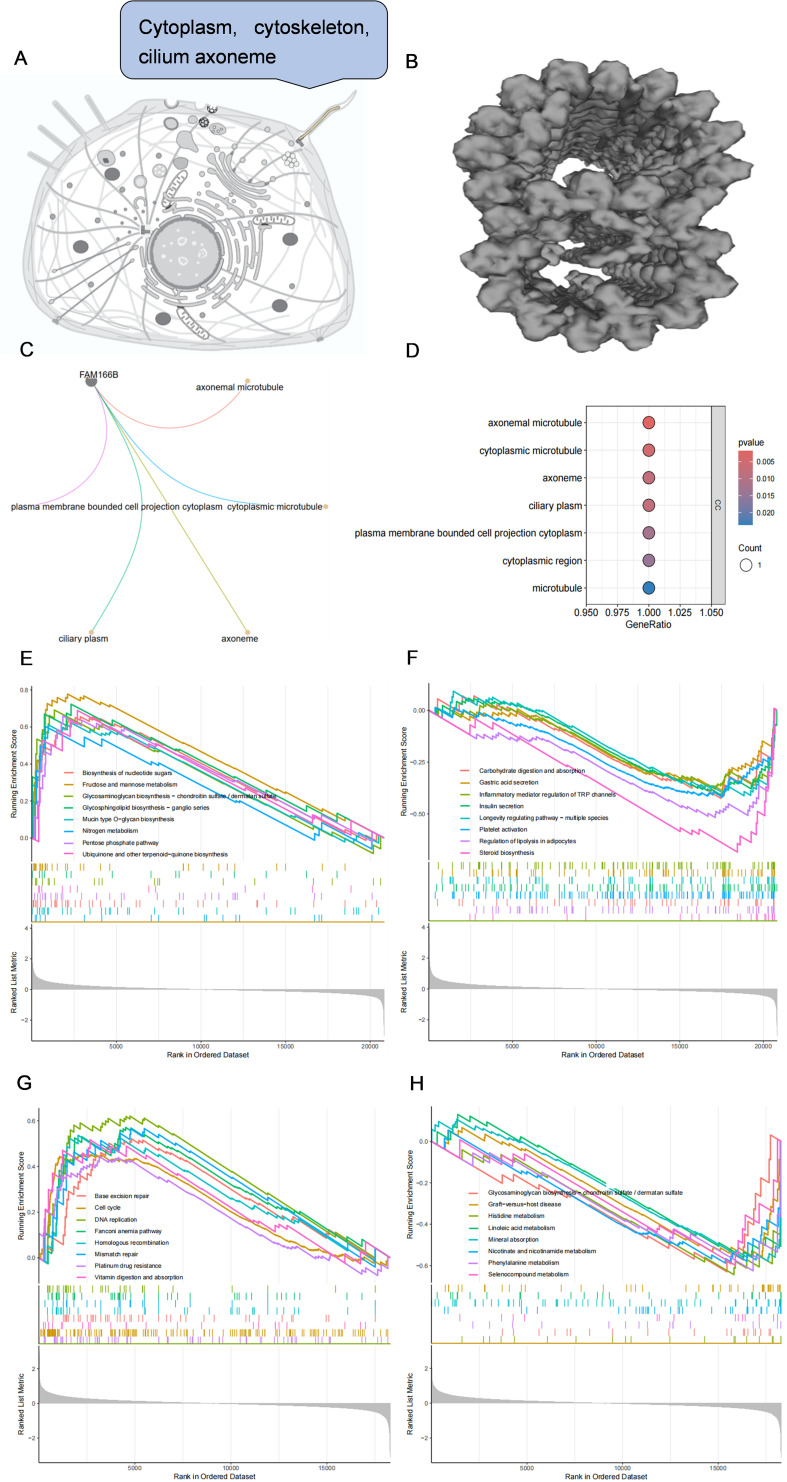
**(A)** Subcellular localization of FAM166B. **(B)** The spatial structure of FAM166B. **(C)** GO enrichment network diagram of FAM166B. **(D)** GO enrichment bubble diagram of FAM166B. **(E)** GSEA up-regulation pathway of FAM166B in PCOS. **(F)** GSEA down-regulation pathway of FAM166B in PCOS. **(G)** GSEA up-regulation pathway of FAM166B in RSA. **(H)** GSEA down-regulation pathway of FAM166B in RSA.

Through the Uniprot online platform, FAM166B was found to be localized in the cytoplasm, cytoskeleton, ciliated axons (**Figure 7A**), and the structure was microtubule-like (**Figure 7B**). In order to further clarify the biological processes in which FAM166B is mainly involved, this study performed GO enrichment and GSEA enrichment analysis on FAM166B. GO enrichment results showed that FAM166 B mainly played an important role in cellular component, and was significantly enriched in axonemal microtubule, cytoplasmic microtubule, axoneme, ciliary plasm and plasma membrane bounded cell projection cytoplasm pathways (**Figures 7C, D**). GSEA enrichment results in PCOS showed that highly expressed FAM166B was mainly enriched in signaling pathways such as Insulin secretion, Fructose and mannose metabolism, Regulation of lipolysis in adipocytes, Inflammatory mediator regulation of TRP channels, Carbohydrate digestion and absorption, Steroid biosynthesis and Platelet activation (**Figures 7E, F**). GSEA enrichment results of RSA showed that highly expressed FAM166B was mainly enriched in signaling pathways such as Histidine metabolism, Phenylalanine metabolism, Linoleic acid metabolism, Nicotinate and nicotinamide metabolism, Cell cycle and DNA replication signaling pathways (**Figures 7G, H**).

### Signal transduction mechanisms associated with co-diagnostic genes

**Figure 8:**
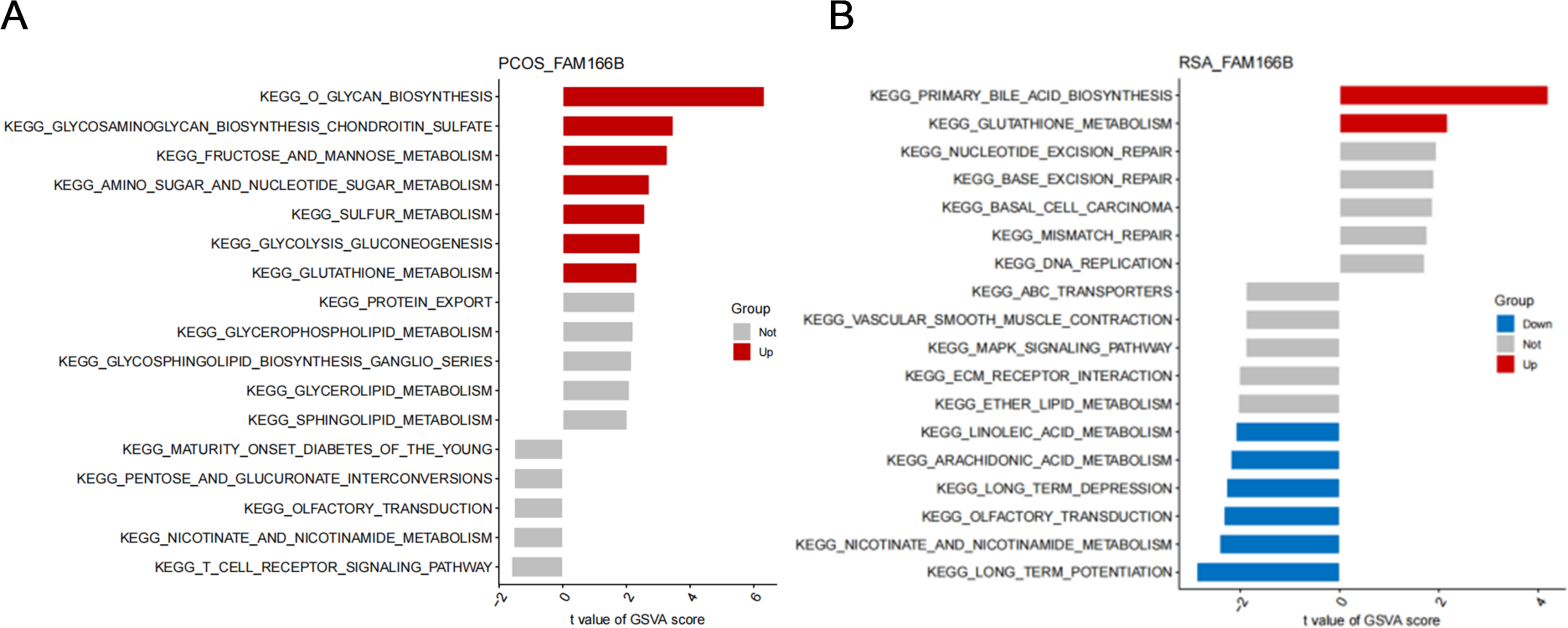
GSVA analysis of high expression of FAM166B. **(A)** GSVA analysis of FAM166B in PCOS. **(B)** GSVA analysis of FAM166B in RSA.

Next, this study explored the effect of FAM166B on disease progression-related signaling pathways. GSVA results of PCOS showed that high expression of FAM166B was mainly enriched for O-glycan biosynthesis, glycosaminoglycan biosynthesis-chondroitin sulfate, fructose and mannose metabolism, Glycolysis/gluconeogenesis, amino sugar and nucleotide sugar metabolism and Glutathione metabolism signaling pathways (**Figure 8A**). GSVA results of RSA showed that up-regulated FAM166B was mainly enriched for primary bile acid biosynthesis and Glutathione metabolism pathways that were significantly up-regulated, and linoleic acid metabolism, Arachidonic acid metabolism, Arachidonic acid metabolism, Nicotinate and nicotinamide metabolism pathways were significantly down-regulated (**Figure 8B**).

### FAM166B and immune cell infiltration

**Figure 9:**
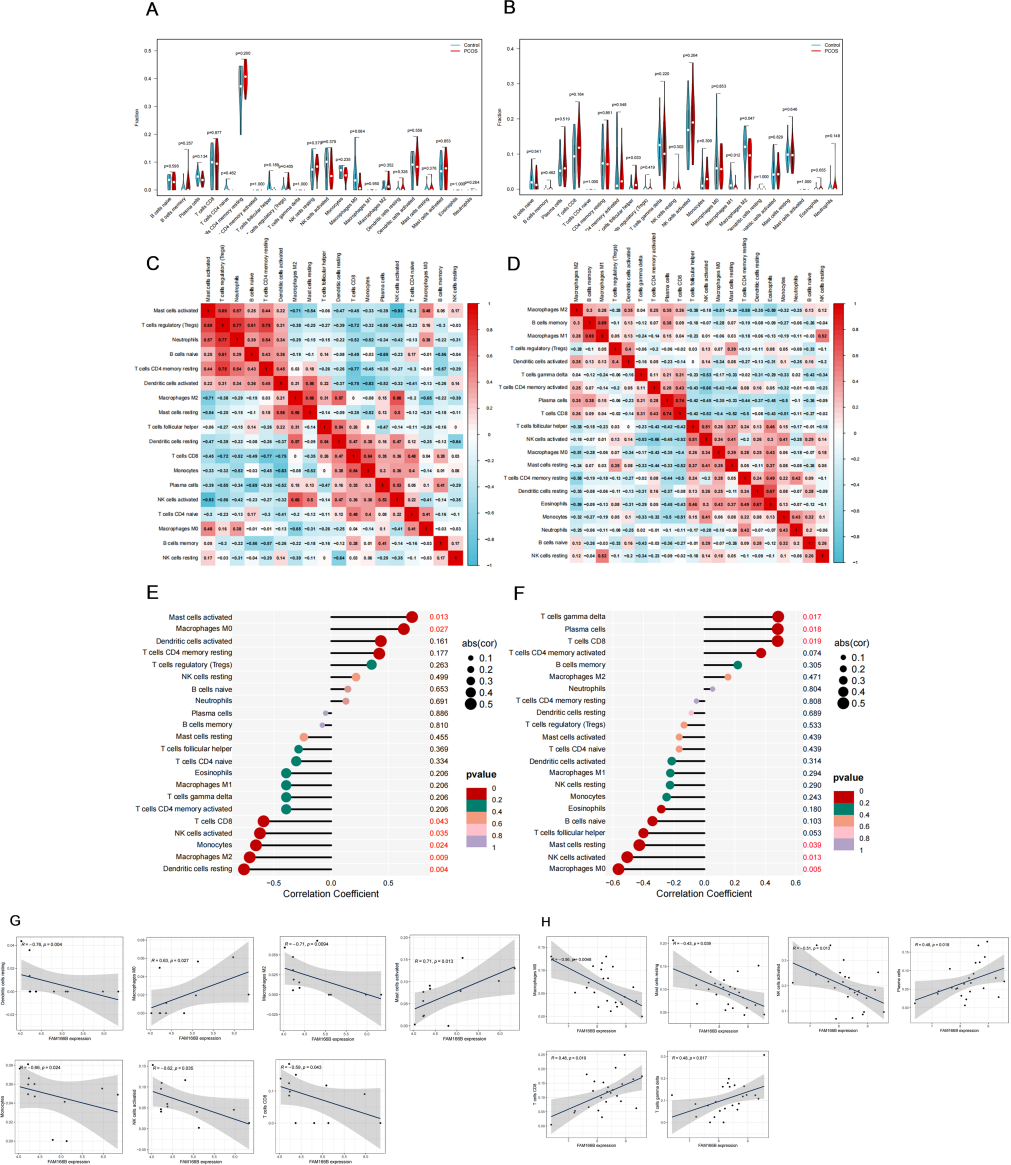
The characteristics of immune cell infiltration and its correlation with shared genes. **(A)** The difference of immune cell infiltration between PCOS and control group. **(B)** The difference of immune cell infiltration between RSA and control group. **(C)** Correlation heat map between 22 kinds of immune cells in PCOS. **(D)** Correlation heat map between 22 kinds of immune cells in RSA. **(E)** Analysis of the correlation between FAM166B and immune cells in PCOS. **(F)** Analysis of the correlation between FAM166B and immune cells in RSA. **(G)** The correlation scatter plot of FAM166 B and immune cells in PCOS. **(H)** The correlation scatter plot of FAM166 B and immune cells in RSA.

Considering that the common feature of PCOS and RSA is that the immune response is more prominent, we used the CIBERSORT algorithm to analyze the distribution of 22 different immune cell types between the disease and the control group and the correlation between shared diagnostic genes and immune cells. As shown in the violin plots, the degree of immune cell infiltration was more significant in RSA than in PCOS (**Figures 9A, B**), in which the degree of T cells follicular helper infiltration was significantly increased in RSA; whereas, the degree of Macrophages M1 and Macrophages M2 infiltration was significantly decreased (**Figure 9B**). Notably, different immune cells interacted with each other and were closely linked (**Figures 9C, D**). In PCOS, FAM166B was positively correlated with Macrophages M0, Mast cells activated; and negatively correlated with Dendritic cells resting, Macrophages M2, Monocytes, NK cells activated and T cells CD8 (**Figures 9E, G**). In the RSA group, FAM166B was positively correlated with T cells gamma delta, Plasma cells, and T cells CD8, and negatively correlated with Macrophages M0, NK cells activated, and Mast cells resting (**Figures 9F, H**), which is in agreement with the former findings (31).

### Human tissue samples were used to verify co-diagnostic genes

**Figure 10:**
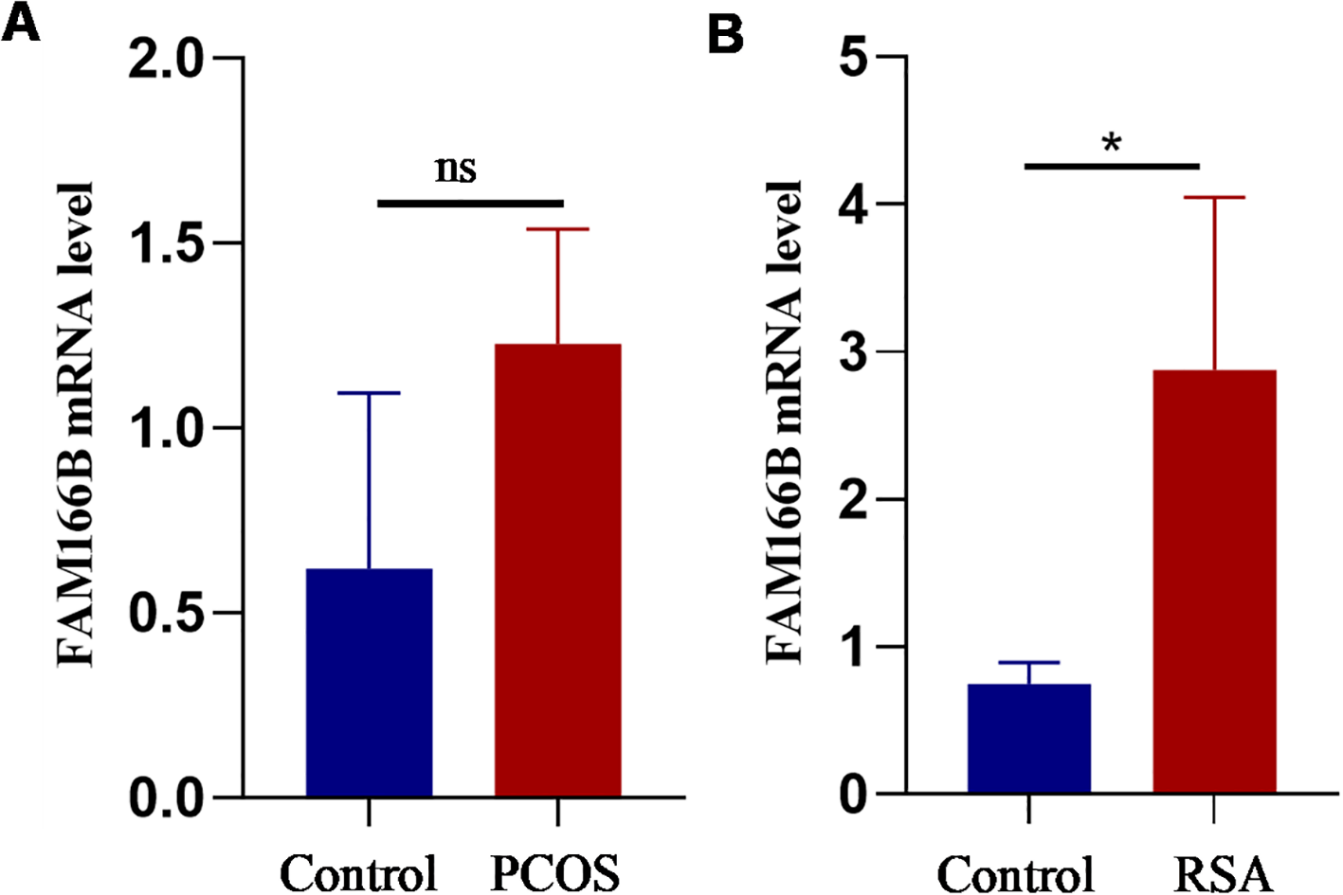
Validation of RT-qPCR in human tissues. **(A)** The expression level of FAM166B in granulosa cells of control and PCOS patients (n = 3). **(B)** The expression level of FAM166 B in chorionic tissues of control and RSA patients (n = 4). ns The difference is not significant, *P < 0.05.

Finally, qRT-PCR analysis was performed in this study in order to validate the expression of FAM166B in granulosa cells and RSA chorionic tissue of PCOS. The results showed that the mRNA expression of FAM166B showed an elevated trend in both PCOS and RSA (**Figures 10A, B**), which was essentially similar to the results of the data analysis described above. Unfortunately, the up-regulation of FAM166B in granulosa cells of PCOS patients was not significant, which may be due to the small sample size.

## Discussion

As a heterogeneous endocrine disease that seriously affects the reproductive health of women of childbearing age, PCOS is affected by a variety of external factors (epigenetics, environmental toxins, physical and emotional stress, diet) and internal factors (insulin resistance, androgen excess, inflammation, oxidative stress, obesity) (32, 33). RSA is a common disease associated with infertility, including embryonic or fetal abortion. Chromosomal abnormalities, reproductive tract abnormalities, immune diseases, endocrine diseases, antiphospholipid syndrome, thrombophilia and pathogen infection have been shown to play an important role in the occurrence of RSA (34). Li et al. showed that decidualization, embryo implantation, trophoblast cell differentiation, invasion and apoptosis, placental development, fetal development, immune response and coagulation process may be involved in the occurrence of RSA (35). It is worth noting that the etiology of about 50% of RSA is not clear, its molecular mechanism is not clear, and the incidence of RSA is increasing year by year (36).

At present, more and more studies have confirmed that there is a close correlation between PCOS and RSA. Cocksedge et al. first used the Rotterdam standard for clinical research and found that the prevalence of PCOS in RSA was 8.3-10% (36–38). However, the common pathological mechanism between PCOS and RSA has not been fully understood. Therefore, actively exploring the molecular mechanism between PCOS and RSA will be the basis for early identification and intervention of the disease.

In this study, the relevant data sets of PCOS and RSA were retrieved based on the GEO database, and a total of 23 shared genes were obtained through a series of bioinformatics analysis. GO enrichment results showed that the shared genes were mainly enriched in the functions of lipid metabolism and G1/S phase transition of cell cycle. This result suggests that the association between PCOS and RSA may be related to lipid metabolism and cell cycle, while previous studies have reported that the occurrence of PCOS or RSA is closely related to lipid metabolism and cell cycle (39–43). DO enrichment results showed that these shared genes were mainly associated with polycystic ovary syndrome, germ cell cancer, germinoma, familial hyperlipidemia and schizoaffective disorder, which further confirmed that FAM166B may be involved in the development of PCOS. KEGG analysis showed that the common genes were mainly enriched in adipocyte lipolysis regulation, prolactin signaling pathway (44, 45), FoxO signaling pathway (46), Hippo signaling pathway (47, 48), Wnt signaling pathway (49–51) and other pathways. The relationship between these pathways and PCOS and RSA has been mentioned in previous studies, but has not been further studied. Subsequently, through three kinds of machine learning and Nomoram identification analysis, a common diagnostic gene FAM166 B was obtained.

FAM166 B is located in the cytoplasm, cytoskeleton and ciliated axons, and the structure is microtubule-like, it is a gene that needs to be further studied (52). Previous studies on FAM166B have shown that it is highly expressed in multiple symmetric lipidosis, skeletal muscle, adrenal gland and ciliated cells (52, 53). Secondly, the expression of FAM166 B is related to the prognosis of breast cancer, and the expression level of FAM166 B in breast cancer is closely related to macrophages and CD4+T cells, which indicates that the recruitment and regulation of immune infiltrating cells in breast cancer may be mediated by FAM166B (54).

In addition, FAM166B may be involved in lymph node metastasis in patients with non-small cell lung cancer (NSCLC) (55). Whereas a recent bioinformatics study demonstrated that FAM166B may be involved in RSA by down-regulating M2-type macrophages, plasma cells, and CD4 resting memory T cells (56), no studies have been found in the pathogenesis of PCOS.

The GO analysis of FAM166 B in this study showed that FAM166 B mainly played an important role in the cellular component, which was significantly enriched in the functions of axonemal microtubule, cytoplasmic microtubule, axoneme, ciliary plasm and plasma membrane bounded cell projection cytoplasm. In GSEA results, FAM166B in PCOS was mainly enriched in signaling pathways such as Insulin secretion, Fructose and mannose metabolism, Regulation of lipolysis in adipocytes, Inflammatory mediator regulation of TRP channels, Carbohydrate digestion and absorption, Steroid biosynthesis and Platelet activation. FAM166B of RSA is mainly enriched in Histidine metabolism, Phenylalanine metabolism, Linoleic acid metabolism, Nicotinate and nicotinamide metabolism, Cell cycle and DNA replication signaling pathways. This suggests that FAM166B may be involved in the development of PCOS or RSA by regulating the cell cycle, amino acid metabolism, lipid metabolism, glucose metabolism, carbohydrate metabolism, and inflammatory responses. Previous studies have shown that PCOS (57) and RSA (58) have different degrees of redox abnormalities. GSVA analysis in this study showed that both PCOS and RSA had severe disorders of glutathione metabolism, which was similar to the results of previous studies.

CIBERSORT-based analysis showed that more severe immune dysfunction existed in RSA than in PCOS, in which the degree of T cells follicular helper infiltration was significantly increased in RSA; whereas the degree of Macrophages M1 and Macrophages M2 infiltration was significantly decreased. In addition, in PCOS, FAM166B was positively correlated with Macrophages M0, Mast cells activated; and negatively correlated with Dendritic cells resting, Macrophages M2, Monocytes, NK cells activated and T cells CD8 Mast cells activated. In the RSA group, FAM166B was positively correlated with T cells gamma delta, Plasma cells, and T cells CD8, and negatively correlated with Macrophages M0, NK cells activated, and Mast cells resting, which is in line with the former findings (31). Zeng and other scholars found that adequate and balanced accumulation of T cells follicular helper during pregnancy may help to maintain a successful pregnancy, while excessively high levels may lead to miscarriage (59). The association between RSA and Macrophages M1 and Macrophages M2 has been reported in previous studies (60).

In summary, FAM166 B may be involved in the occurrence and development of PCOS and RSA by changing the cell cycle, oxidative stress, immune microenvironment and so on. The newly discovered diagnostic genes and potential molecular mechanisms in this study provide new clinical insights and guidance for the diagnosis and treatment of PCOS and RSA patients.

Although this study combines WGCNA, LASSO model, RF algorithm and SVM-RFE algorithm to identify common potential biomarkers associated with the pathogenesis of PCOS and RSA, and there is no joint analysis of these two diseases. However, this study still has some limitations. First, our current study only involves one diagnostic gene; secondly, the sample size is relatively small, and future research needs larger sample size and more predictive clinical indicators to verify these results. Thirdly, the research on the related mechanism of PCOS and RSA is not deep enough. If proteomics, metabolomics and microbiome can be combined and analyzed, the physiological mechanism of the two diseases will be better understood.

## Conclusions

Our study preliminarily revealed the common potential genes and molecular mechanisms closely related to PCOS and RSA. Among them, the FAM166 B gene may have a critical impact on the pathophysiological mechanisms of PCOS and RSA and may be involved in the development and progression of the disease by altering the cell cycle, oxidative stress status, and immune microenvironment. These findings may help to develop early diagnostic strategies, prognostic markers and therapeutic targets.

## Data Availability

Publicly available datasets used in this study can be downloaded from the GEO database (http://www.ncbi.nlm.nih.gov/geo/). The PCOS group includes three datasets with GSE10946 and GSE6798 and GSE137684. GSE165004 and GSE26787 and GSE22490 are involved in the RSA group. If necessary, please contact the corresponding author to obtain the original data.
