# Supplementary material for: Shared diagnostic genes and potential mechanisms between polycystic ovary syndrome and recurrent miscarriage revealed by integrated transcriptomics analysis and machine learning
Source: Front Endocrinol (Lausanne). 2024 Sep 27;15:1335106. doi: 10.3389/fendo.2024.1335106 (PMC11466764; doi:10.3389/fendo.2024.1335106)
Supplement: Supplementary file 1 [file Table1.docx]

**Supplementary Table 1** Name and sequence of primers in RT-qRCR

| Gene | Primer sequences（5'-3') |
| --- | --- |
| Human GAPDH | F：GGAAGCTTGTCATCAATGGAAATC |
|  | R：TGATGACCCTTTTGGCTCCC |
| Human CIMIP2B | F：TCAGCTCCAGCATGATCCCT |
|  | R：TTTCTTCCCTTGGCCTCCTT |
